# Postoperative Monocyte Count Change Is a Better Predictor of Survival Than Preoperative Monocyte Count in Esophageal Squamous Cell Carcinoma

**DOI:** 10.1155/2019/2702719

**Published:** 2019-08-14

**Authors:** Qian Song, Jun-zhou Wu, Sheng Wang

**Affiliations:** ^1^Department of Clinical Laboratory, Institute of Cancer Research and Basic Medical Sciences of Chinese Academy of Sciences, Cancer Hospital of University of Chinese Academy of Sciences, Zhejiang Cancer Hospital, Hangzhou, Zhejiang, China; ^2^Cancer Research Institute, Institute of Cancer Research and Basic Medical Sciences of Chinese Academy of Sciences, Cancer Hospital of University of Chinese Academy of Sciences, Zhejiang Cancer Hospital & Key Laboratory Diagnosis and Treatment Technology on Thoracic Oncology of Zhejiang Province, Hangzhou, Zhejiang, China

## Abstract

In esophageal squamous cell carcinoma, an elevated preoperative absolute monocyte count (Pre-AMC) is reported to be a predictor of survival, but the clinical application of postoperative absolute monocyte count change (AMCc) remains unknown. The present study was designed to investigate the prognostic value of AMCc in ESCC. 686 patients of ESCC after radical surgery without preoperative adjuvant therapy were enrolled. The Pre-AMC and AMCc were recorded within one week before the operation and one week after surgery. We considered the median of Pre-AMC as the optimal cut-off value to evaluate the relationship between Pre-AMC and patient survival. AMCc was defined as AMCc increased (higher than Pre-AMC) and AMCc decreased (lower than Pre-AMC). Demographic and clinical characteristics, disease-free survival (DFS), and overall survival (OS) were statistically analyzed. Multivariate analysis revealed that AMCc was a better independent prognostic factor for both OS (P = 0.002, HR = 0.614, 95% CI 0.450-0.837) and DFS (P = 0.023, HR = 0.656, 95% CI 0.456-0.943) than Pre-AMC which was only an independent prognostic factor for OS (P = 0.033, HR = 2.031, 95% CI 1.058-3.898). AMCc could be a better prognostic factor than Pre-AMC in patients with ESCC. AMCc decrease predicts worse OS and DFS in ESCC undergoing curative resection.

## 1. Introduction

Worldwide, esophageal cancer ranks seventh in incidence with 572,000 new cases and sixth in terms of mortality with 509,000 deaths, which suggests that this disease will be responsible for an estimated 1 in every 20 cancer deaths by 2018. In China, this disease ranks fifth in terms of incidence and fourth in mortality overall [1]. Esophageal squamous cell carcinoma usually comprises over 90% of all esophageal cancer cases in China [2, 3]. With the development of multimodality therapies, however, patients with ESCC still face with worse prognosis [4, 5]. Several factors including TNM stage and tumor differentiation have been reported that have relationship with the prognosis of ESCC. Nevertheless, patients with the same TNM stage have inconsistent prognosis [6]. Therefore, it is urgent to investigate new and suitable prognosis biomarkers.

A growing number of evidence indicates that inflammation might play a critical role in carcinogenesis of cancer [7, 8]. Many systemic inflammation based factors including tumor-associated macrophages (TAMs) and neutrophil-to-lymphocyte ratio (NLR) are independent prognostic factors of various cancers [9–12]. TAMs are key regulators of the tumor microenvironment and derived from myeloid progenitor cells and monocytes [13].

Several studies have shown that an increased preoperative absolute monocyte count (Pre-AMC) could predict unfavorable survival in patients with various carcinomas, such as lung adenocarcinoma [14], hepatocellular carcinoma [15], prostate cancer [16] and esophageal cancer [17]. These studies focused primarily on Pre-AMC, while the dynamic change of absolute monocyte count (AMC) after therapy was not considered. The postoperative absolute monocyte count change (AMCc) might be a meaningful parameter to assess survival after therapy, because the therapy including surgery and chemotherapy could cause a change. However, the AMCc might dynamically reflect the systemic inflammatory response against cancer after therapy, its significance is unknown. Therefore, this retrospective study aimed to investigate the association between AMCc and clinical features, and to evaluate the prognostic value of AMCc in patients with ESCC.

## 2. Results

### 2.1. Patient Characteristics

We enrolled 686 patients with ESCC in the present research, including 583 (85.0%) males and 103 (15.0%) females. The median age at therapy initiation was 61 years (range from 39 to 84 years). The median value of Pre-AMC was 0.5 (range from 0.2 to 1.6). We chose the median value of Pre-AMC as the cut-off value. One week after curative resection, the median value of postoperative AMC (Post-AMC) was 0.7 (range from 0.1 to 2.1). When comparing the Pre-AMC and Post-AMC, AMCc was increased in 573 (83.5%) patients and decreased in 113 (16.5%) patients. The 1-year OS and 1-year DFS of all patients were 48.3%, 46.2%. The 3-year OS and 3-year DFS of all patients were 25.7%, 24.5%. In addition, the 5-year OS and 5-year DFS were 12.1%, 11.4%. The baseline characteristics of patients with ESCC in the two AMCc groups were shown in Table 1. There was no significant difference between the two groups in the baseline features, except the females were more (P = 0.045), the median of Pre-AMC was lower (P <0.001) and the median of Post-AMC was higher (P <0.001) in AMCc increased group than in AMCc decreased group.

### 2.2. Differences in Overall Survival and Disease-Free Survival according to AMCc

**Figure 1:**
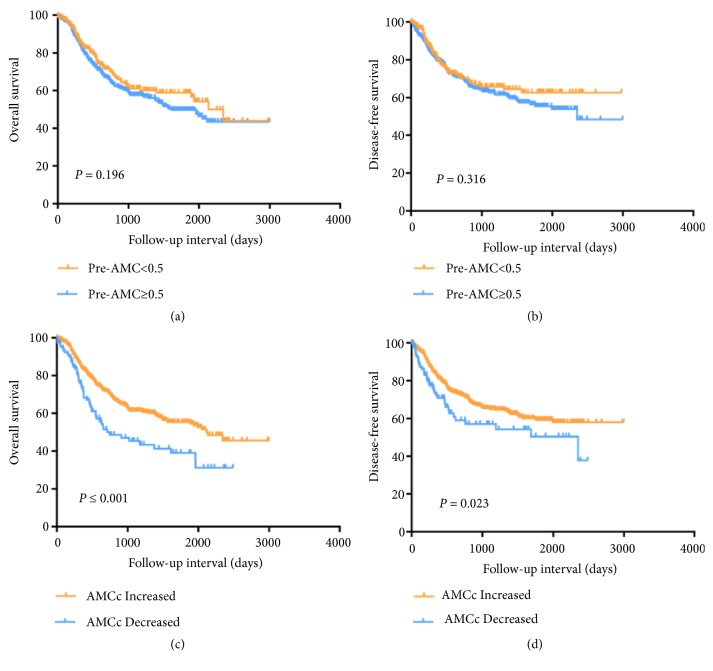
Overall survival and disease-free survival analysis according the preoperative absolute monocyte count (Pre-AMC) (a, b) and postoperative absolute monocyte count change (c, d).

We chose the median of Pre-AMC as the cutoff value and divided the patients into low Pre-AMC group (Pre-AMC <0.5) and high Pre-AMC group (Pre-AMC ≥0.5). The Pre-AMC was significantly associated with overall survival (OS) (HR, 2.145; 95% CI, 1.124-4.095; P = 0.021), but not to disease-free survival (DFS) (HR, 1.853; 95% CI, 0.883-3.889; P = 0.103). After adjustment for confounders, there was significant relationship between Pre-AMC and OS (HR, 2.031; 95% CI, 1.058-3.898; P = 0.033), but not to DFS (Tables 2 and 3). As shown in Figure 1, the Kaplan–Meier curves indicated that there was no significant difference between low Pre-AMC group and high Pre-AMC group both in OS (P=0.196, Figure 1(a)) and DFS (P=0.316, Figure 1(b)).

In univariate analyses of AMCc, there was a significant difference in OS (HR, 0.581; 95% CI, 0.428-0.789; P = 0.001) and DFS (HR, 0.671; 95% CI, 0.468-0.962; P = 0.030) between the AMCc increased group and AMCc decreased group. In multivariate analyses, AMCc decreased was associated with worse OS (HR, 0.614; 95% CI, 0.450-0.837; P = 0.002) and DFS (HR, 0.656; 95% CI, 0.456-0.943; P = 0.023) (Tables 4 and 5). The Kaplan–Meier curves suggested that AMCc decreased could be predict worse OS (P⩽0.001, Figure 1(c)) and DFS (P=0.023, Figure 1(d)). Above all, AMCc was a better independent prognostic factor than Pre-AMC in patients with ESCC after esophageal radical surgery.

## 3. Discussion

The systemic inflammatory reaction in the tumor microenvironment plays a critical part in tumorigenesis and progression [18]. The systemic inflammatory reaction increases the circulating counts of monocytes, neutrophils and platelets [19–21]. Preoperative absolute monocyte count (Pre-AMC) has been demonstrated to be associated with the prognosis of various cancers. However, all studies reported the role of Pre-AMC without the evaluation of the AMCc. These researches focused only on the Pre-AMC, not on the dynamic changes in AMC after therapy. Moreover, no universal cut-off value of Pre-AMC exists, which was typically set by the receiver operating characteristics curve (ROC) [14], or mean value [22]. Therefore, the optimal cut-off value of Pre-AMC varied in different researches, no matter there were the real variations between different clinical laboratories and races [14–17]. We think that an optimal cut-off value of Pre-AMC or Post-AMC that satisfies all of the clinical laboratories in worldwide does not exist. In this study, for the first time, we evaluated AMCc that was not affected by the cut-off value of AMC and might reflect the dynamic change of the systemic inflammatory reaction from preoperative to postoperative. We demonstrated that AMCc could be a better prognosis factor in patients with ESCC after curative resection than Pre-AMC.

In this study, multivariate analysis indicated that AMCc was an independent prognosis biomarker both for OS and DFS. While Pre-AMC was only an independent prognosis biomarker for OS, not for DFS. This findings were inconsistent with the previous study, which reported Pre-AMC could predict worse outcomes both in OS and DFS in patients with ESCC [17]. The reason for this inconsistent was that we used different manners to choose optimal cut-off value, and that different numbers of patients were enrolled. Interestingly, when comparing the demographic and clinical features of the AMCc increased and decreased group, we found that the median Pre-AMC was lower and the median Post-AMC was higher in the AMCc increased group than AMCc decreased group. This finding suggested that the balance between the systemic inflammatory reaction and immune reaction might change after the curative surgery. This change might lead to a different prognosis. However, the precise mechanism that monocyte might predict clinical suivival are not fully understood. One proposed postulation is as follows: monocytes are recruited by some cytokines and chemokines around the cancer. Then monocytes differentiate into tumor-associated macrophages (TAMs), which facilitate numerous pro-tumorigenic mechanisms. Macrophages are classified into the two states: the classically activated type 1 macrophages (M1) and the alternatively activated type 2 macrophages (M2) [23]. M1 produces type I proinflammatory cytokines, present antigen to T cells for an adaptive immune response, and partake anti-tumor function. While M2 produces type II cytokines and contributes to pro-tumorigenic effect including tumor initiation, invasion, angiogenesis and metastasis [24, 25]. TAM infiltration was related to overall worse outcome and poor responses to chemotherapy in patients with ESCC [26–28]. In the present study, AMCc increased is significantly associated with worse outcome in ESCC. When the relationship between systemic inflammatory reaction and immune reaction is out of balance, this might partake the pro-tumor function that leads to worse prognosis. AMCc could accurately represent the dynamic change of the relationship between systemic inflammatory reaction and immune reaction from preoperative to postoperative.

There were several limitations of this study: first, it was the single-center design and retrospective analysis. Multi-center design and prospective trials are needed to prove these findings. Second, our data were not divided into a training set and a validation set for statistical validation. In the future, we are looking forward to the similar results of other types of cancer.

## 4. Methods

### 4.1. Patient Selection

748 patients newly diagnosed with ESCC from Feb 2008 to Feb 2015 at the Zhejiang Cancer Hospital were enrolled in this study. The World Health Organization classification criteria were the standard for the determination of the histological grades. These included patients were pathologically confirmed, and received operation after diagnosis without preoperative adjuvant therapy including chemotherapy or radiotherapy. There are 62 patients were excluded: 45 patients with preoperative chemotherapy, 9 patients with preoperative radiotherapy, 3 patients without Pre-AMC available, and 5 patients without Post-AMC available. As a result, 686 patients with ESCC were chosen in the present study.

The Pre-AMC was examined within 1 week prior to surgery, and the Post-AMC was checked after 1 week after curative operation. The present study was approved by the Ethics Committee of Zhejiang Cancer Hospital. All included patients completed written informed consent.

### 4.2. Blood-Routine Markers

Blood (2 mL) was drawn into the EDTA-K2 anticoagulative tubes for a routine fasting blood sample. The peripheral monocyte and platelet were checked by the SYSMEX XE-2100 (Sysmex, Kobe, Japan) Automatic Blood Cell Analyzer.

### 4.3. Statistical Analysis

The Pre-AMC, Post-AMC, Pre-platelet and Post-platelet were analyzed as continuous variables and all clinical features were counted as categorical variables. Categorical data are expressed as numbers and percentage, and continuous data, which do not meet a normal distribution, are presented as median and interquartile range. The relationship between AMCc and clinical features of ESCC was analyzed using chi-square tests. The median of Pre-AMC was chosen to determine the optimal cut-off value. The Kaplan-Meier method was shown overall survival (OS) and disease-free survival (DFS). The Kaplan-Meier curve was analyzed by GraphPad Prism 7 software. The effect of clinical features on prognosis was calculated by the log-rank test. Univariate and multivariate COX regression analyses were used to evaluate the predictors, which were expressed as hazard ratio, 95% confidence interval and P value. P <0.05 was considered to be statistically significant. The SPSS software (version 19.0) was used for statistical analysis.

## Figures and Tables

**Table 1:** Demographic and clinical data of 686 ESCC patients accroding to AMC change.

Charateristics		Total (N=686), %	AMCc		*P *value
			Increased (N=573), %	Decreased (N=113), %	
Sex	Male	583 (85.0)	480 (83.8)	103 (91.2)	*0.045*
	Female	103 (15.0)	93 (16.2)	10 (8.8)	
Age (years)	⩽60	316 (46.1)	270 (47.1)	46 (40.7)	0.211
	>60	370 (53.9)	303 (52.9)	67 (59.3)	
Pathology grade	Well differentiated	49 (7.1)	44 (7.7)	5 (4.4)	0.335
	middle differentiated	454 (66.2)	381 (66.5)	73 (64.6)	
	Poorly differentiated	167 (24.3)	133 (23.2)	34 (30.1)	
	Undifferentiated	2 (0.3)	2 (0.3)	0 (0)	
	Missing	14 (2.0)	13 (2.3)	1 (0.9)	
Depth of tumor	T1a–1b	64 (9.3)	53 (9.2)	11 (9.7)	0.983
	T2	132 (19.2)	110 (19.2)	22 (19.5)	
	T3	490 (71.4)	410 (71.6)	80 (70.8)	
Lymph node metastasis	N0	298 (43.4)	245 (42.8)	53 (46.9)	0.686
	N1	215 (31.3)	185 (32.3)	30 (26.5)	
	N2	119 (17.3)	98 (17.1)	21 (18.6)	
	N3	54 (7.9)	45 (7.9)	9 (8.0)	
Pathological stage	1a–1b	117 (17.1)	94 (16.4)	23 (20.4)	0.565
	2a–2b	230 (33.5)	195 (34.0)	35 (31.0)	
	3a–3c	339 (49.4)	284 (49.6)	55 (48.7)	
Vessel invasive	Yes	210 (30.6)	173 (30.2)	37 (32.7)	0.591
	No	476 (69.4)	400 (69.8)	76 (67.3)	
Nerve infiltration	Yes	256 (37.3)	208 (36.3)	48 (42.5)	0.215
	No	430 (62.7)	365 (63.7)	65 (57.5)	
Treatment regimen	S	464 (67.6)	384 (67.0)	80 (70.8)	0.710
	S + postoperative C	157 (22.9)	133 (23.2)	24 (21.2)	
	S + postoperative CRT	65 (9.5)	56 (9.8)	9 (8.0)	
Hospital time (days)	⩽14	542 (79.0)	460 (80.3)	82 (72.6)	0.066
	>14	144 (21.0)	113 (19.7)	31 (27.4)	
Pre-AMC	Median	0.5 (0.4-0.6)	0.5 (0.4-0.6)	0.7 (0.5-0.9)	*<0.001*
Post-AMC	Median	0.7 (0.6-0.9)	0.8 (0.6-1.0)	0.5 (0.3-0.6)	*<0.001*
Pre-Platelet	Median	201 (160-238)	206.5 (149.5-244.0)	200.0 (162.8-237.3)	0.500
Post-Platelet	Median	261 (200-331)	263.0 (202.0-332.3)	251.5 (177.0-324.0)	0.074

S, surgery; C, chemotherapy; CRT, chemoradiotherapy; Pre-AMC: preoperative absolute monocyte count; Post-AMC: postoperative absolute monocyte count; AMCc: postoperative absolute monocyte count change; Pre-platelet: preoperative platelet; Post-platelet: postoperative platelet.

**Table 2:** Overall survival analyses according to Pre-AMC in 686 patients with ESCC.

Variables	Univariate			Multivariate		
	HR	95% CI	*P* value	HR	95% CI	*P* value
Pre-AMC	2.145	1.124-4.095	*0.021*	2.031	1.058-3.898	*0.033*
Sex	1.175	0.809-1.705	0.398			
Age (years)	1.001	0.984-1.018	0.926			
Depth of tumor	1.508	1.177-1.932	*0.001*	1.344	0.970-1.862	0.075
Lymph node metastasis	1.758	1.548-1.996	*<0.001*	1.777	1.449-2.180	*<0.001*
Pathological stage	1.905	1.548-2.344	*<0.001*	0.813	0.561-1.179	0.275
Vessel invasive	1.753	1.348-2.280	*<0.001*	1.186	0.892-1.576	0.24
Nerve infiltration	1.838	1.422-2.376	*<0.001*	1.543	1.177-2.023	*0.002*
Treatment regimen	1.009	0.899-1.131	0.883			
Hospital time (days)	1.007	0.995-1.019	0.24			

**Table 3:** Disease-free survival analyses according to Pre-AMC in 686 patients with ESCC.

Variables	Univariate			Multivariate		
	HR	95% CI	*P* value	HR	95% CI	*P* value
Pre-AMC	1.853	0.883-3.889	0.103			
Sex	1.177	0.778-1.782	0.440			
Age (years)	0.993	0.9974-1.011	0.425			
Depth of tumor	1.137	0.898-1.439	0.286			
Lymph node metastasis	1.601	1.382-1.855	*<0.001*	1.596	1.283-1.987	*<0.001*
Pathological stage	1.557	1.257-1.928	*<0.001*	0.825	0.603-1.131	0.232
Vessel invasive	1.273	0.935-1.732	0.125			
Nerve infiltration	1.608	1.205-2.145	*0.001*	1.506	1.113-2.036	*0.008*
Treatment regimen	1.368	1.219-1.535	*<0.001*	1.264	1.118-1.428	*<0.001*
Hospital time (days)	0.998	0.982-1.013	0.759			

**Table 4:** Overall survival analyses according to AMC change in 686 patients with ESCC.

Variables	Univariate			Multivariate		
	HR	95% CI	*P* value	HR	95% CI	*P* value
AMCc (increased vs. decreased)	0.581	0.428-0.789	*0.001*	0.614	0.450-0.837	*0.002*
Sex	1.175	0.809-1.705	0.398			
Age (years)	1.001	0.984-1.018	0.926			
Depth of tumor	1.508	1.177-1.932	*0.001*	1.339	0.970-1.849	0.076
Lymph node metastasis	1.758	1.548-1.996	*<0.001*	1.766	1.441-2.165	*<0.001*
Pathological stage	1.905	1.548-2.344	*<0.001*	0.838	0.578-1.217	0.354
Vessel invasive	1.753	1.348-2.280	*<0.001*	1.184	0.892-1.573	0.243
Nerve infiltration	1.838	1.422-2.376	*<0.001*	1.479	1.129-1.936	*0.004*
Treatment regimen	1.009	0.899-1.131	0.883			
Hospital time (days)	1.007	0.995-1.019	0.24			

**Table 5:** Disease-free survival analyses according to AMC change in 686 patients with ESCC.

Variables	Univariate			Multivariate		
	HR	95% CI	*P* value	HR	95% CI	*P* value
AMCc (increased vs. decreased)	0.671	0.468-0.962	*0.030*	0.656	0.456-0.943	*0.023*
Sex	1.177	0.778-1.782	0.440			
Age (years)	0.993	0.9974-1.011	0.425			
Depth of tumor	1.137	0.898-1.439	0.286			
Lymph node metastasis	1.601	1.382-1.855	*<0.001*	1.572	1.263-1.957	*<0.001*
Pathological stage	1.557	1.257-1.928	*<0.001*	0.849	0.620-1.165	0.311
Vessel invasive	1.273	0.935-1.732	0.125			
Nerve infiltration	1.608	1.205-2.145	*0.001*	1.462	1.081-1.978	*0.014*
Treatment regimen	1.368	1.219-1.535	*<0.001*	1.274	1.128-1.440	*<0.001*
Hospital time (days)	0.998	0.982-1.013	0.759			

## Data Availability

The data used to support the findings of this study are available from the corresponding author upon request.
